# Acupuncture to Reduce HIV-Associated Inflammation

**DOI:** 10.1155/2015/908538

**Published:** 2015-04-02

**Authors:** Barbara Swanson, Joyce K. Keithley, Angela Johnson, Louis Fogg, Oluwatoyin Adeyemi, Beverly E. Sha, Kimberly A. Snell

**Affiliations:** ^1^Rush University College of Nursing, 600 South Paulina, Suite 1080, Chicago, IL 60612, USA; ^2^Cancer Integrative Medicine Program, Rush University Medical Center, Chicago, IL 60612, USA; ^3^CORE Center, Cook County Bureau of Health, Chicago, IL 60612, USA; ^4^Section of Infectious Diseases, Rush University Medical Center, Chicago, IL 60612, USA; ^5^Rush University Medical Center, Chicago, IL 60612, USA

## Abstract

*Background*. HIV infection is associated with systemic inflammation that can increase risk for cardiovascular events. Acupuncture has been shown to have immunomodulatory effects and to improve symptoms in persons with inflammatory conditions.* Objective*. To test the anti-inflammatory effects of an acupuncture protocol that targets the cholinergic anti-inflammatory pathway (CAIP), a neural mechanism whose activation has been shown to reduce the release of proinflammatory cytokines, in persons with HIV-associated inflammation.* Design, Setting, Participants, and Interventions*. Double-blind, placebo-controlled clinical trial conducted in an outpatient clinic located in a medically underserved urban neighborhood. Twenty-five clinically-stable HIV-infected persons on antiretroviral therapy were randomized to receive once weekly CAIP-based acupuncture or sham acupuncture.* Main Outcome Measures*. Outcomes included plasma concentrations of high sensitivity C-reactive protein and D-dimer and fasting lipids.* Results*. Twenty-five participants completed the protocol (treatment group *n* = 12, control group *n* = 13). No adverse events related to the acupuncture protocol were observed. Compared to baseline values, the two groups did not significantly differ in any outcome measures at the end of the acupuncture protocol.* Conclusions*. CAIP-based acupuncture did not favorably modulate inflammatory or lipid parameters. Additional studies are warranted of CAIP-based protocols of different frequencies/durations.

## 1. Introduction

Persistent elevation of inflammatory markers is common in HIV-infected persons and has been associated with the development of dyslipidemia and cardiovascular events, as well as all-cause mortality [1, 2]. While there is no accepted standard of care to reduce HIV-related inflammation, a small body of literature suggests that statins and cyclooxygenase (COX) inhibitors have limited or modest effectiveness for favorably modulating soluble markers of inflammation and coagulation [3–5]. In the absence of proven pharmacological therapies, there is a need for alternative therapies that have the potential for reducing persistent inflammation in HIV-infected persons. One alternative is acupuncture, which has been shown to have immunomodulatory effects [6] and to improve symptoms in persons with inflammatory conditions [7, 8]. Thus it is plausible that using acupuncture to target anti-inflammatory pathways, such as the cholinergic anti-inflammatory pathway (CAIP), may inhibit the release of proinflammatory cytokines and reduce cardiovascular risk [9]. The CAIP is a neural mechanism that inhibits the release of proinflammatory cytokines and minimizes the deleterious effects of inflammation [10]. However, to date, there have been no animal or human studies that have tested the anti-inflammatory effects of acupuncture that targets the CAIP. The purpose of this randomized placebo-controlled clinical trial was to explore the effects of an eight-week, once weekly acupuncture protocol that specifically targets the CAIP on soluble markers of inflammation and plasma lipoproteins.

## 2. Materials and Methods

The study used a randomized, placebo-controlled, double-blind clinical trial design to evaluate the efficacy and safety of an 8-week course of CAIP-based acupuncture in persons with HIV-related inflammation. Participants were randomized 1 : 1 to the CAIP-based or placebo conditions using computer-generated random numbers. Data were collected at baseline and at the eighth acupuncture session. All personnel, except the statistician and acupuncturist, were blinded to participant assignment.

### 2.1. Setting and Sample

The study was conducted between July 2011 and October 2012 at two outpatient HIV/AIDS clinics located in a federally designated medically underserved neighborhood in Chicago. Institutional review board approval was granted and the ethical standards of the Helsinki Declaration of 1975 were followed. All participants gave written informed consent before enrollment into the study.

Participants were eligible if they were HIV-infected adults between 18 and 60 years of age with an hsCRP level of ≥3.0 mg/L, a CD4 T cell count of at least 250 cells/mm^3^, and a viral load of <75 copies/mL and had been on stable antiretroviral therapy for at least two months.

Participants were excluded if they had a history of heart attack or stroke; had a platelet count <150,000 cells/mm^3^; had a chronic condition known to affect lipid status (i.e., diabetes mellitus, familial hyperlipidemia); had a chronic inflammatory condition (i.e., rheumatoid arthritis, inflammatory bowel disease, and systemic lupus erythematosus); used within the previous month medications or herbal therapies reported to influence bleeding or inflammatory parameters (e.g., COX inhibitors, steroids or other immunosuppressive medications, aspirin >100 mg/day, ginseng, and glucosamine/chondroitin); ever received acupuncture; had lymphedema in any body part; had documented active opportunistic infections or malignancies; had serum creatinine >2.0 mg/dL or liver enzyme elevations >three times the upper limit of normal; has body mass index ≥30; used lipid-lowering medications; or were currently using illicit drugs or consuming ≥three alcoholic drinks/day.

### 2.2. Procedures

Participants were recruited via flyers and outreach efforts by the clinics' research coordinators. Participants completed a total of 10 study visits. At the first two visits (one to two weeks apart), medical history data and blood samples were collected to determine eligibility. At the third visit (one to two weeks later), participants completed a Traditional Chinese Medicine (TCM) history form after which the acupuncturist performed a TCM assessment and assigned a TCM diagnosis. The acupuncturist then opened the randomization envelope and introduced the participant to the concept of needle insertion using either actual acupuncture needles (for participants randomized to the CAIP-based group) or the placebo needles (for participants randomized to the placebo group). The acupuncturist then performed the assigned 30-minute acupuncture treatment. Participants returned to the clinic for seven additional visits to receive the assigned 30-minute acupuncture treatment. We selected this protocol because 6–8 weeks of acupuncture treatment has been shown to reduce symptoms in inflammatory conditions, such as arthritis [11] and prostatitis [12], while once weekly acupuncture has been associated with improvements in knee osteoarthritis [13, 14].

Acupuncture treatments were performed by two licensed acupuncturists who were certified in “Clean Needle Technique” by the Council of Colleges of Acupuncture and Oriental Medicine (CCAOM). To promote treatment fidelity, both acupuncturists followed a procedure manual that detailed the steps in the TCM assessment and the two acupuncture protocols.

### 2.3. Intervention

To control for the effects of social interaction on the study outcomes, the acupuncturist's conversation with the participant was limited to treatment-specific issues. The acupuncturist swabbed each acupuncture point location with an alcohol swab and inserted the needles. Throughout the 30-minute treatment period, the acupuncturist returned at 10-minute intervals to check on the participant. At the end of 30 minutes, the needles were removed. Two acupuncture protocols were delivered: CAIP-based and placebo.

#### 2.3.1. CAIP-Based Protocol

Each acupuncture treatment was delivered using disposable, sterile, stainless steel 36-gauge Serin needles (DongBang Acuprime, Exeter, UK). The acupuncturist placed a single needle bilaterally at the following four points: (1) LU1; (2) ST 36; (3) PC6; and (4) LI4. These points have been shown to correspond to vagal stimulation [15–25]. Each needle was manually stimulated until the patient experienced the characteristic* de-Qi* needle sensation. This procedure, including point selection, was repeated at each of the eight acupuncture sessions.

#### 2.3.2. Placebo Protocol

Participants randomized to the placebo group did not receive real acupuncture. Instead, the acupuncturist used a needle called a “Park Sham Device” (DongBang Acuprime, Exeter, UK) that does not penetrate the skin because it is encased in a cartridge [26]. The cartridge prevents the participant from knowing whether the needle has been inserted or not. This is a preferred method to sham acupuncture, where a needle is inserted and minimally manipulated, because (a) placebo acupuncture does not actually penetrate the skin and is thus considered physiologically inert and (b) studies have shown that acupuncture-naïve participants cannot distinguish true penetrating acupuncture from placebo acupuncture [26, 27]. The Park Sham Device needles were placed at the same four sites that were used for the CAIP-based protocol.

### 2.4. Measures

All primary and secondary outcome measurements were performed at a CLIA-certified commercial laboratory (Labcorp, Elmhurst, IL).

#### 2.4.1. Primary Outcomes

High sensitivity C-reactive protein (hsCRP) concentration was measured using a latex immunoturbidimetry assay. Due to biological variability, two baseline measurements of hsCRP were obtained and the mean was used in the analyses. Plasma concentration of D-dimer was quantified at the baseline and final study visits using an immunoturbidimetry assay. We chose these markers because elevated serum levels of hsCRP and D-dimer are indicative of systemic inflammation, are common in HIV infection [28], and are highly related to all-cause mortality in persons with HIV/AIDS [2].

#### 2.4.2. Secondary Outcomes

The standard lipid profile was obtained following an overnight fast at the baseline and final study visits and consisted of triglycerides and total, high-density lipoprotein (HDL), and low-density lipoprotein (LDL) cholesterol. Lipids were quantified using spectrophotometric methods. Safety parameters were measured at the baseline and final study visits. CD4+ T lymphocytes were quantified using two-color (anti-CD3/anti-CD4) flow cytometry and plasma HIV-1 RNA was quantified using a polymerase chain reaction assay.

### 2.5. Data Analysis

Descriptive statistics were used to characterize the groups' demographic and clinical data. Independent samples *t*-tests and chi-square analyses were used to test for baseline group differences. For each outcome, the differences in the two groups' change scores (each participant's value at the final visit minus value at baseline) were determined and independent samples *t*-tests were performed. Effect sizes (Cohen's D) for the CAIP-based acupuncture were calculated by dividing the treatment group's change score for each inflammatory and lipid outcome by the standard deviation for that change score.

## 3. Results

**Figure 1:**
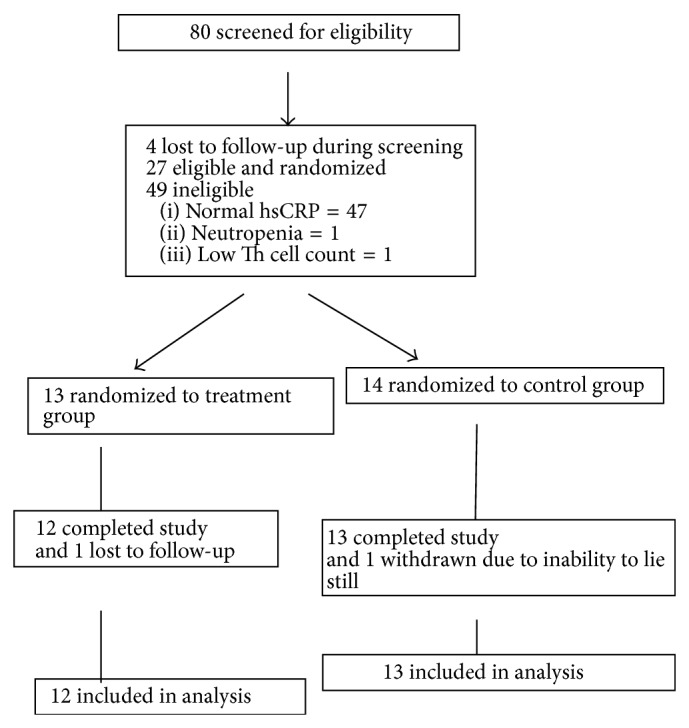
Study design and flow diagram.

A total of 80 participants were screened for eligibility, 27 were randomized and entered into the analyses, and 25 completed the protocol (Table 1, Figure 1). Most of the ineligible participants did not meet the study's hsCRP criteria (≥3.0 mg/L). Baseline demographic and clinical data of the participants who completed the protocol are shown in Table 2. The groups did not significantly differ on any baseline demographic, clinical, or outcome measures (all *P* values > .5).

No statistically significant differences were found between the groups' change scores for any of the primary or secondary outcomes (Tables 3 and 4). Effect size analyses showed that CAIP-based acupuncture had a negligible effect for favorably modulating inflammatory and lipid parameters (Table 4). No participants reported any side effects related to either the CAIP-based or sham acupuncture protocols. Both CD4 counts and plasma concentration of HIV RNA remained stable over the study period.

## 4. Discussion

This pilot study is the first to explore the safety and efficacy of an eight-week CAIP-based acupuncture protocol to favorably modulate inflammatory and lipid parameters in adults with HIV-related inflammation. While the acupuncture protocol was well-tolerated, we found no significant differences between the two groups on any outcome measures and all effect sizes were in the low range (Cohen's *d* range = .11 to .45).

We selected acupuncture points that corresponded to vagal output in the TCM paradigm and had additionally been found to modulate markers of vagal output in healthy humans, such as heart rate and heart rate variability (P6, LI4, and LU1) [15, 23, 24] and gastric acid secretion (ST 36) [22]. However, because we did not measure markers of vagal output, we cannot confirm that the acupuncture protocol directly stimulated the vagus as we hypothesized. It is also possible that the duration and/or frequency of treatments was inadequate to induce durable anti-inflammatory effects. We selected the eight-week duration because previous studies found improvement in inflammatory conditions, such as arthritis [11] and prostatitis [12], after 6 to 8 weeks of acupuncture treatment. However, HIV infection may be characterized by a higher inflammatory burden than other inflammatory conditions; thus longer-duration and higher-frequency protocols may be required.

Measurement issues may have contributed to a type II error. It is possible that anti-inflammatory effects were present immediately after the completion of each acupuncture session but had subsided by the time of the posttreatment measurements. Potential lack of sensitivity of our outcome measures may have precluded detecting treatment effects. We have previously shown that nuclear magnetic resonance- (NMR-) derived lipoprotein particle profiles are more sensitive than the standard lipid profile for identifying HIV-infected persons at risk for coronary heart disease [29]. Additionally, both hsCRP and D-dimer are characterized by high intraindividual variability which may obscure efficacy estimates of anti-inflammatory treatments [30, 31]. Other measures of lipids and inflammation may be more sensitive markers of the effects of acupuncture than the measures used in this study. These measures may include NMR-derived lipoprotein particle profiles and intracellular cytokine concentrations.

## 5. Conclusions

Our findings suggest that CAIP-based acupuncture is feasible and not associated with adverse events when administered to clinically-stable HIV-infected adults with evidence of systemic inflammation. Additional studies with larger samples, more sensitive measures of inflammation and lipid metabolism, and objective measures of vagal activation are needed to determine if CAIP-based acupuncture is efficacious for reducing inflammation and/or normalizing lipid profiles.

## Figures and Tables

**Table 1:** Description of screened participants (*N* = 80).

Gender	
(i) Male	51
(ii) Female	29
Age	47.65 (SD = 7.1)
Ethnicity	
(i) Black, non-Hispanic	77
(ii) Hispanic	2
(iii) White	1

**Table 2:** Description of participants at baseline.

	Control group (*n* = 13) Mean (SD)	Treatment group (*n* = 12) Mean (SD)	*P* value
Gender (male/female)	9/4	8/4	.891
Age (years)	47.69 (4.8)	47.33 (9.1)	.902
Ethnicity (Black/Hispanic/White)	12/1/0	11/0/1	.367
CD4 count (cells/mm^3^)	649 (276)	928 (557)	.137
CD4 range (cells/mm^3^)	372 to 1079	353 to 2295	NA
HIV RNA (copies/mL)	44.92 (129)	38.9 (112)	.903
hsCRP (mg/L)	5.37 (2.4)	9.1 (7.1)	.100
D-dimer (*μ*g FEU/mL)	0.42 (0.19)	0.40 (0.41)	.872
Total cholesterol (mg/dL)	187 (39)	187 (35)	.987
LDL cholesterol (mg/dL)	109 (24)	116 (33)	.539
HDL cholesterol (mg/dL)	50 (25)	47 (10)	.746
Triglycerides (mg/dL)	142 (59)	117 (45)	.256

**Table 3:** Change in mean CD4 count and mean HIV RNA from baseline to eighth acupuncture session.

	Control group (*n* = 13)	Treatment group (*n* = 12)	*t* score; *P* value; Cohen's *d*
CD4 change	−20.5 cells/mm^3^ (SD = 101.1)	+28.9 cells/mm^3^ (SD = 272.3)	−0.593; *P* = .563; *d* = .11
HIV RNA change	−14.5 copies (SD = 27.9)	−31.6 copies (SD = 112)	.532; *P* = .60; *d* = −.28

**Table 4:** Change in hsCRP, D-dimer, and lipids from baseline to eighth acupuncture session.

	Control group (*n* = 13)	Treatment group (*n* = 12)	*t* score; *P* value; Cohen's *d*
hsCRP change^1^	6.66 mg/L (SD = 24.4)	−3.19 mg/L (SD = 8.0)	1.32; *P* = .201; *d* = .27
D-dimer change^2^	−0.04 *μ*g FEU/mL (SD = 0.18)	0.03 *μ*g FEU/mL (SD = 0.13)	−.913; *P* = .375; *d* = −.22
Total cholesterol change	−11.42 mg/dL (SD = 25.4)	3.46 mg/dL (SD = 23.4)	−1.52; *P* = .142; *d* = −.45
LDL-cholesterol change	−3.38 mg/dL (SD = 24.9)	4.96 mg/dL (SD = 19.3)	−.929; *P* = .363; *d* = .14
HDL-cholesterol change	−3.5 mg/dL (SD = 8.9)	−1.92 mg/dL (SD = 9.1)	−.442; *P* = .663; *d* = −.39
Triglycerides change	−21.9 mg/dL (SD = 67.2)	1.79 mg/dL (SD = 25.8)	−1.53; *P* = .141; *d* = −.33

^1^Due to missing data, control group *n* = 10.

^2^Due to missing data, control group *n* = 11 and treatment group *n* = 7.
